# Detection of *Apicystis bombi* (Apicomplexa: Neogregarinorida) in carpenter bees of Argentina

**DOI:** 10.1016/j.ijppaw.2023.03.008

**Published:** 2023-04-07

**Authors:** Santiago Plischuk, Silvina Quintana, Gregorio Fernandez De Landa, Pablo Damián Revainera, Marina Haramboure, Carlos Ernesto Lange

**Affiliations:** a Centro de Estudios Parasitológicos y de Vectores (CEPAVE) (CONICET-UNLP), La Plata, Argentina; b Instituto de Investigaciones en Producción, Sanidad y Ambiente - IIPROSAM (CONICET-UNMDP), Mar del Plata, Argentina; c Consejo Nacional de Investigaciones Científicas y Técnicas (CONICET), Argentina; d Agronutris, Saint Orens de Gameville, France; e Comisión de Investigaciones Científicas de la Provincia de Buenos Aires (CIC-PBA), Argentina

**Keywords:** *Apicystis cryptica*, Pollinator, Species introduction, *Xylocopa*

## Abstract

Historically, the neogregarine *Apicystis bombi* was isolated almost exclusively from bumble bees (*Bombus* spp.) where it disrupts adipose tissue, increasing hosts’ mortality rates. Records in solitary bees are scarce worldwide. To check for its presence in carpenter bees (genus *Xylocopa*), campaigns were performed in Argentina capturing 154 individuals of five species (*X. augusti*, *X. splendidula*, *X. atamisquensis*, *X. frontalis*, *X. nigrocincta*). The presence of *A. bombi* was detected by molecular means in *X. augusti*, *X. atamisquensis,* and *X. nigrocincta* in four of the nine provinces screened. The pathogenesis and eventual impact that *A. bombi* may cause in individuals or populations of *Xylocopa* species remain unknown. The presence of *A. bombi* in northern Argentina would be contradictory to the hypothesis that its occurrence is the exclusive result of its introduction to South America through invasive, infected exotic bumble bees.

## Highlights

• *Xylocopa* individuals were scrutinized for the neogregarine *Apicystis bombi*.
• *Apicystis bombi* was found in *X. augusti*, *X. atamisquensis* and *X. nigrocincta*.
• The new associations *Xylocopa* – *A. bombi* were found in four Argentine provinces.

## Graphical abstract

**Figure:**
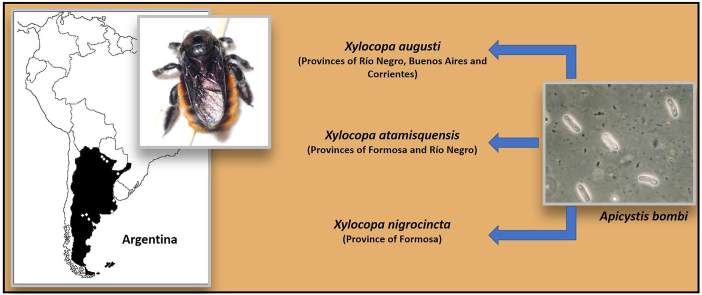

## 1 Introduction

As a group, neogregarines (Apicomplexa: Neogregarinorida) are among the most virulent protists affecting insects (Lange and Lord, 2012). The neogregarine *Apicystis bombi* was originally described as *Mattesia bombi* in association with eight species of bumble bees (*Bombus* spp.) (Liu et al., 1974). Lipa and Triggiani (1992) detected it in both the buff-tailed bumble bee *Bombus terrestris* and the European honey bee *Apis mellifera*, and later they transferred it to the newly erected genus *Apicystis* (Lipa and Triggiani, 1996). Since then, *A. bombi* was registered almost exclusively in bumble bees causing disruption of the adipose tissue as well as nutritional imbalances, increasing mortality rates (Rutrecht and Brown, 2008). During the last decade, however, infections in other bees (Apidae: Meliponini, Megachilidae, Andrenidae) have been reported (Ravoet et al., 2014; Nunes Silva et al., 2016), although knowledge about associated pathologies with them is still incipient (Tian et al., 2018).

The carpenter bees (*Xylocopa* spp.; Apidae: Xylocopini) are solitary,^1^ polylectic insects that visit economically relevant plant species (e.g.: *Passiflora edulis* Sims [Passion fruit], *Vaccinium corymbosum* L. [Blueberry], *Solanum melongena* L. [Eggplant] (Ospina, 2000; Lucia, 2011; Alvarez et al., 2014). Species of the genus get their name of “carpenters” because they excavate galleries and nest in hard wood, usually dead (Ospina, 2000). Regarding parasitic natural enemies associated with the 18 species of carpenter bees known to be present in Argentina, only records of conopids (Diptera: Conopidae) and viruses have been reported in association with six and five of them, respectively (Stuke et al., 2011; Lucia et al., 2014; Quintana et al., 2019; Fernández de Landa et al., 2020).

Since host-switch phenomena between bee species have been increasingly discovered as well as novel infection pathways using by different parasites and pathogens (Koch et al., 2017; Figueroa et al., 2023), our aim was to screen an understudied and ecologically important genus of bees in Argentina for *Apicystis*. Specifically, we screened five species of *Xylocopa* sampled in nine provinces from 2009 to 2019 for *A. bombi* and *A. cryptica*, showing information about the presence, geographic distribution, and new host species of the former. We also briefly discuss about the potential risks that this parasite-host complex might represent for the country's apiculture, as well as why the detection of *A. bombi* in northern Argentina would not be compatible with the hypothesis that its occurrence in South America was exclusively as an outcome of the introduction of infected exotic bumble bees (see Arbetman et al., 2013).

## 2 Materials and methods

**Fig. 1:**
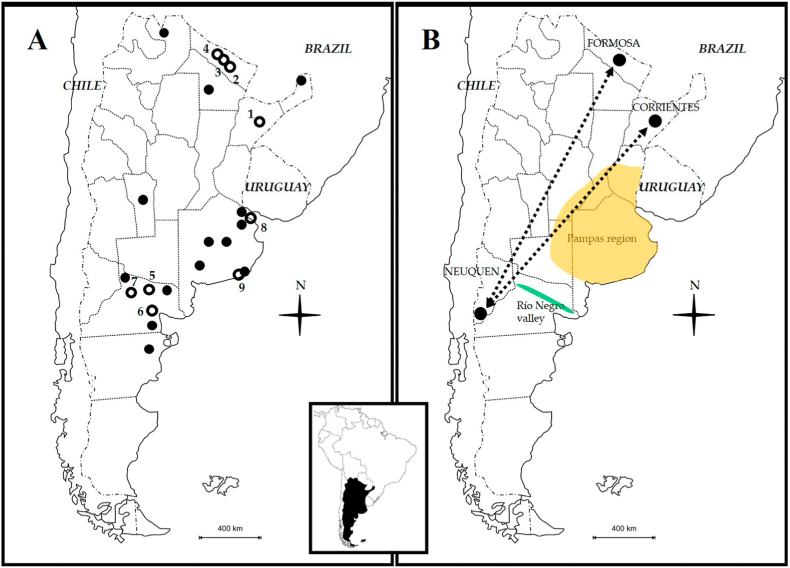
A. Sampled sites. Solid circles: localities with detections of *Apicystis bombi*. Empty circles: Localities without detections; number of each locality corresponds to those detailed in Table 1. **B.** Distance (dotted arrows, ∼2000 km) between Corrientes-Formosa provinces and Neuquén province (see main text). Shade areas show Pampas region (yellow) and Río Negro valley (green). (For interpretation of the references to colour in this figure legend, the reader is referred to the Web version of this article.)

As part of a project on pollinator pathologies in the country, a total of 152 female adult carpenter bees [*Xylocopa augusti* (n = 89), *X. splendidula* (n = 31), *X. atamisquensis* (n = 21), *X. frontalis* (n = 6), and *X. nigrocincta* (n = 5)] were captured in natural (protected areas), rural roadsides and agricultural lands from 29 localities (nine provinces) of Argentina throughout Spring-Summer seasons between 2009 and 2019 (Fig. 1A and see Table 1 on Electronic supplementary material for details). Individuals were collected by walking slowly through a site and captured either directly into plastic vials or by netting. Since our objective was to obtain an initial glance on the presence and diversity of *Apicystis* in these pollinators, we prioritized to carry out collections over large areas rather than large numbers of individuals *per* site. Therefore, due to the limited number of bees *per* site, some epizootiological parameters such as prevalence are not included as they are not statistically representative. After collecting, bees were kept alive in a cooler, identified (Lucia, 2011), and stored in absolute ethanol.

**Table 1:** *Apicystis bombi* detections in carpenter bees (*Xylocopa* spp.) of Argentina.

Province	Locality (Reference to Fig. 1A)	Coordinates	Host species
Corrientes	Colonia Pellegrini (1)	28°32′17″ S	*X. augusti*
		57°11′12″ W	
Formosa	Palo Santo (2)	25°33′59″ S	*X. nigrocincta*
		59°16′53″ W	
	Ibarreta (3)	25°14′07″ S	*X. atamisquensis*
		59°48′19″ W	
	Las Lomitas (4)	24°32′59″ S	*X. atamisquensis*
		60°27′41″ W	
Río Negro	Villa Regina (5)	39°05′22″ S	*X. augusti*
		67°05′07″ W	
	General Conesa (6)	40°04′50″ S	*X. atamisquensis*
		64°28′23″ W	
	General Roca (7)	39°01′02″ S	*X. augusti*
		67°36′59″ W	
Buenos Aires	Arana (8)	35°00′28″ S	*X. augusti*
		57°54′46″ W	
	Mar del Plata (9)	37°56′01″ S	*X. augusti*
		57°40′41″ W	

After individual homogenization, total genomic DNA was extracted by means of a High Pure PCR Template Preparation kit (Roche Diagnostics) and amplified by qPCR using two pair of primers: ApBF1 and ApBR1 - which targets *A. bombi* as well as *Apicystis cryptica* DNA, plus ApC500f and ApC666r 5 - which targets only *A. cryptica* DNA (Schoonvaere et al., 2020) (see Electronic supplementary material for details). DNA fragments obtained with ApBF1 and ApBR1 primers were purified and directly sequenced (ABI 3500 Genetic Analyzer, Applied Biosystems, Foster City, CA, USA). The sequences similarities were determined by the Basic Local Alignment Search Tool (BLAST, NCBI).

## 3 Results and discussion

Presence of *A. bombi* was detected in nine individuals of *Xylocopa* spp.: five specimens of *X. augusti* collected in the provinces of Río Negro (localities of Villa Regina and General Roca), Buenos Aires (Arana and Mar del Plata), and Corrientes (Colonia Pellegrini), three *X. atamisquensis* [two from Formosa province (Ibarreta and Las Lomitas) and one from Río Negro (General Conesa)], and one *X. nigrocincta* captured in Formosa (Palo Santo) (Fig. 1A, Table 1). Sequencing confirmed that isolates belonged to *A. bombi.* Two of the isolates were double-sequenced and deposited in Genbank as follows: NCBI accession number MT547746 corresponds to Villa Regina (Río Negro) sampled from *X. augusti*, and MT547735 to Palo Santo (Formosa) sampled from *X. nigrocincta*.

At the time of collection, the bees did not show any evident sign or symptom of disease [*e.g.,* sluggishness, disorientation, inability/difficulty for flying (Lange and Lord, 2012)], and as the first natural record of this host-parasite complex, the effects that *A. bombi* could produce on *Xylocopa* species were not yet recognized. Since *Bombus* species and, at least experimentally, *Osmia bicornis* (Megachilidae), a solitary bee, seem to be seriously affected by *A. bombi*, a similar virulence in *Xylocopa* species might be possible, yet remains to be determined (MacFarlane et al., 1995; Tian et al., 2018).

Regarding detection areas, the Río Negro valley (Fig. 1B -green shade-) has more than 50,000 ha under fruit production, especially apple (*Malus domestica* Borkh) and pear (*Pyrus communis* L.) (Avellá et al., 2018). Presence of bumble bees there is not usual; instead, occurrence of carpenter bees is relatively common (Abrahamovich et al., 2007; Lucia, 2011). It is likely that carpenter bees (and possibly *A. mellifera*) play a leading role as pollinating agents and occupy an ecological niche in the region similar to that of *Bombus* (Lucia, 2011). Therefore, as closely related organisms, it is conceivable that *Bombus* spp. and *Xylocopa* spp. may share part of their parasitic niche, as it was seen with the fungus *Ascosphaera apis* (Gilliam et al., 1994), various viruses [DWV (Lucia et al., 2014), AmFV (Quintana et al., 2019), CBPV (Fernández de Landa et al., 2020)], and even dipteran parasitoids (Stuke et al., 2011; Plischuk et al., 2018). At the population level, the impact of a pathogen on non-social species would be more relevant than on social ones, since affecting a potentially fertile female implies affecting not only her but all her offspring (Tian et al., 2018). Pathogens, also, could modify the foraging behavior of affected bees (see Koch et al., 2017). Then once the areas where *A. bombi* is present has been identified, the focus should be on deciphering how and to what extent this gregarine is affecting the *Xylocopa* populations.

The detection of *A. bombi* in the northern provinces of Corrientes (Colonia Pellegrini) and Formosa (Ibarreta and Palo Santo) (Fig. 1b) turns out to be an enigmatic outcome. Two exotic, Palearctic bumble bees (*Bombus ruderatus* and *B. terrestris*) were introduced in Chile for pollination purposes in 1982 and 1998, respectively, becoming established and expanding in both Chilean and Argentine Patagonia region (Smith-Ramírez et al., 2018). It has been hypothesized that they (or one of them) would have carried *A. bombi* upon entering South America and triggered spillover and host-switch events while interacting with native pollinator species (see Arbetman et al., 2013). Since both *B. terrestris* and *B. ruderatus* seem to be absent in the transition areas [nearby a *ca*. 2000 km-long axis (Fig. 1b, arrow)] (see Aizen et al., 2019), the arrival of *A. bombi* at Corrientes - Formosa somehow after its ingress in southern Neuquén province is difficult to explain. Although a dispersal of this pathogen through other host species cannot be ruled out, the possibility that *A. bombi* was present in South America prior to the introduction of exotic bumble bees may be also plausible. Previous findings of *A. bombi* in Colombia (Gamboa et al., 2015) and Brazil (Nunes Silva et al., 2016) would support such a possibility. Furthermore, DNA of *A. bombi* has been found in museum specimens collected *ca*. 1950, strongly suggesting its presence in Argentina prior to the introduction events in South America (Revainera et al., 2020).

Finally, the Pampas region is located in the center-east of Argentina and contains more than one million honey bee hives that generate almost half of the total production in the country (Food and Agriculture Organization Statistical Database, 2019). Detections of *A. bombi* in Río Negro valley (northern Patagonia), where many beekeepers bring transhumant hives from the Pampas region (Ugalde and García-Sartor, 2015), could represent a source of inoculum potentially transmissible to honey bees by sharing floral resources with *Xylocopa* (Koch et al., 2017), and return to their origin at the end of season. Sanitary status of these hives should be closely monitored to avoid a potential spread of *A. bombi* in the Pampas.

## Declaration of competing interest

None.

## Footnotes

1 Although some species exhibit some degree of sociability (see Lucia, 2011).
