# Supplementary material for: Detection of Apicystis bombi (Apicomplexa: Neogregarinorida) in carpenter bees of Argentina
Source: Int J Parasitol Parasites Wildl. 2023 Apr 7;21:43–6. doi: 10.1016/j.ijppaw.2023.03.008 (PMC10133405; doi:10.1016/j.ijppaw.2023.03.008)
Supplement: Multimedia component 1 [file mmc1.pdf]

IJPPAW - Electronic Supplementary Material

Detection of *Apicystis bombi* (Apicomplexa: Neogregarinorida) in carpenter bees of Argentina

Santiago PLISCHUK, Silvina QUINTANA, Gregorio FERNANDEZ DE LANDA, Pablo Damián REVAINERA, Marina HARAMBOURE & Carlos Ernesto LANGE

Molecular analyses

Each bee was homogenized and total genomic DNA was extracted using a High Pure PCR Template Preparation kit (Roche Diagnostics). To verify the success of DNA extraction, an amplification of a 134 bp PCR product of an internal reference control DNA (5´-AGATGGGGGCATTCGTATTG-3´, 5´-ATCTGATCGCCTTCGAACCT-3´) was performed (Nunes-Silva et al. 2016).

The cycling program consisted of an initial denaturation of 2 minutes at 95 °C, and 40 cycles of 94 °C (20 sec), 52 °C (20 sec), 72 °C (30 sec). After amplification, a melting curve analysis was performed, which resulted in single product-specific melting curve. Those samples with 18S rDNA Ct (Cycle Threshold) values < 35 were considered suitable.

In order to study the presence of *Apicystis bombi* we performed qPCR amplification with primers ApBF1 F CGTACTGCCCTGAATACTCCAG and ApBR1 R TGAAAGCGGCGTATACATGA which amplified a 293 bp PCR product (Meuss et al., 2010). The cycling program consisted of an initial denaturation of 2 minutes at 95 °C, and 40 cycles of 95 °C (20 sec), annealing temperature of 58 °C (20 sec), 72 °C (30 sec). Samples with Ct values < 30 were considered positive. The specific dissociation

temperature of specific *A. bombi* PCR product was 82 °C. The reaction efficiency for this primer pair was 98%, as calculated by analysing ten-fold dilutions of pure neogregarine DNA (Burd, 2010). During the validation process the PCR products were run on agarose gels to check the size of the PCR products. All qPCR reactions were carried out in a Rotor Gene Q thermocycler (Qiagen, Hilden, Germany) in a final volume of 20 µl using EvaGreen as intercalating fluorescent dye (KAPA FAST, Biosystems, Woburn, USA). In each qPCR run positive, negative and non-template controls were added. As positive controls, pure DNA extracted from this neogregarine was used. Negative controls consisted in DNA from samples in which the absence of the pathogen's DNA was confirmed previously.

To verify the specificity of the selected primers, amplified DNA fragments were purified and directly sequenced (ABI 3500 Genetic Analyzer, Applied Biosystems, Foster City, CA, USA). The sequences similarities were determined by the Basic Local Alignment Search Tool (BLAST, NCBI).

We also tested to amplify positive samples with primers ApC500f 5 -CAACCCCATGCAAGTATCAA and reverse ApC666r 5 -TGCCAATAAAAACAAAGCTCAA, described as specific to *Apicystis cryptica* DNA (Schoonvaere et al., 2020) and no amplification was obtained, verifying that *A. bombi* DNA was present in our samples.

Table 1. Detail of samplings. Total of screened bees (n), showing species, location, year, and individuals with *Apicystis bombi* (n+).

Species	Province	Locality	n	n+	Year
Xylocopa atamisquensis	Chubut	Sierra grande	2	-	2018
	Formosa	Ibarreta	2	1	2012
		Las Lomitas	2	1	2015
	Río Negro	Belisle	1	-	2014
		General Conesa	2	1	2018
		General Roca	8	-	2014
	Salta	Río Seco	2	-	2011
	San Luis	La Carolina	2	-	2012
Xylocopa augusti	Buenos Aires	Arana	1	-	2009
			2	-	2010
			1	-	2013
			3	1	2015
		Azul	8	-	2016
		Bolívar	2	-	2012
		Buenos Aires	1	-	2012
		City Bell	6	-	2010
			2	-	2015
			3	-	2016
			2	-	2017
		Colonia Urquiza	3	-	2010
			1	-	2012
			1	-	2014
		La Plata	2	-	2009
			8	-	2011
			2	-	2013
			1	-	2016
		Lobos	1	-	2009
		Lomas de Zamora	1	-	2016
		Mar del Plata	10	1	2019
		Pearson	3	-	2014
			1	-	2016
			2	-	2017
		Punta Lara	1	-	2009
		Villa Ventana	2	-	2016
	Corrientes	Colonia Pellegrini	1	1	2009
	Río Negro	Allen	1	-	2011
		General Roca	12	1	2016
		Villa Regina	5	1	2014

Table 1. Detail of samplings (cont.). Total of screened bees (n), showing species, location, year, and individuals with *Apicystis bombi* (n+).

Species	Province	Locality	n	n+	Year
Xylocopa frontalis	Buenos Aires	Isla Martin García	1	-	2009
		La Plata	1	-	2010
			2	-	2011
			2	-	2016
Xylocopa nigrocincta	Chaco	Tres estacas	1	-	2011
	Formosa	Palo Santo	3	1	2012
	Misiones	Puerto Iguazú	1	-	2015
Xylocopa splendidula	Buenos Aires	City Bell	1	-	2016
		Colonia Urquiza	1	-	2013
		La Plata	2	-	2010
			1	-	2016
		Punta Lara	1	-	2016
	Chubut	Gaiman	24	-	2016
	Río Negro	Belisle	1	-	2014

References

Burd, E.M., 2010. Validation of Laboratory-Developed Molecular Assays for Infectious Diseases. Clin. Microbiol. Rev. 23(3), 550-576. 10.1128/CMR.00074-09

Meeus, I., De Graaf, D.C., Jans, K., Smagghe, G., 2010. Multiplex PCR detection of slowly-evolving trypanosomatids and neogregarines in bumblebees using broad-range primers. J. App. Microbiol. 109(1), 107-115. 10.1111/j.1365-2672.2009.04635.x

Nunes-Silva, P., Piot, N., Meeus, I., Blochtein, B., Smagghe, G., 2016. Absence of Leishmaniinae and Nosematidae in stingless bees. Sci. Rep. 6, 32547. 10.1038/srep32547
